# Physicochemical compatibility of caffeine citrate and caffeine base injections with parenteral medications used in neonatal intensive care settings

**DOI:** 10.1007/s00228-024-03678-6

**Published:** 2024-03-28

**Authors:** D. Thisuri N. De Silva, Michael Petrovski, Tobias Strunk, Nabeelah Mukadam, Madhu Page-Sharp, Brioni R. Moore, Kevin T. Batty

**Affiliations:** 1 Curtin Medical School, Curtin University, Bentley, WA Australia; 2 Pharmacy Department, Sir Charles Gardiner Hospital, North Metropolitan Health Service, Nedlands, WA Australia; 3 Medical School, The University of Western Australia, Crawley, WA Australia; 4 Wesfarmers Centre for Vaccines and Infectious Diseases, Telethon Kids Institute, Nedlands, WA Australia; 5 Neonatal Directorate, King Edward Memorial Hospital, Child and Adolescent Health Service, Subiaco, WA Australia; 6 Pharmacy Department, King Edward Memorial Hospital, Women and Newborn Health Service, Subiaco, WA Australia; 7 Curtin Health Innovation Research Institute, Curtin University, Bentley, WA Australia

**Keywords:** Caffeine citrate, Caffeine base, Physical compatibility, Chemical compatibility, Neonates

## Abstract

**Purpose:**

To investigate the physicochemical compatibility of caffeine citrate and caffeine base injections with 43 secondary intravenous (IV) drugs used in Neonatal Intensive Care Unit (NICU) settings.

**Methods:**

Caffeine citrate (20 mg/mL or 10 mg/mL) or caffeine base injection (10 mg/mL) were mixed in a volume ratio of 1:1 with the secondary drug solution to simulate Y-site co-administration procedures in NICUs. Physical compatibility was evaluated based on visual observation for 2 h, against a black and white background and under polarised light, for changes in colour, precipitation, haze and evolution of gas. Chemical compatibility was determined from caffeine concentration measurements, using a validated high-performance liquid chromatography assay.

**Results:**

Six of the 43 secondary drugs tested (aciclovir, amphotericin (liposomal), furosemide, hydrocortisone, ibuprofen and ibuprofen lysine) were physically incompatible with caffeine citrate undiluted injection (20 mg/mL), at their high-end, clinically relevant concentrations for NICU settings. However, when tested at lower concentrations, hydrocortisone (1 mg/mL) was physicochemically compatible, whereas furosemide (0.2 mg/mL) was physically incompatible with caffeine citrate. The six drugs which showed physical incompatibility with caffeine citrate 20 mg/mL injection were also physically incompatible with caffeine citrate 10 mg/mL solution. All 43 secondary drugs tested were physicochemically compatible with caffeine base injection.

**Conclusions:**

Most secondary test drugs, except aciclovir, amphotericin (liposomal), furosemide, hydrocortisone, ibuprofen and ibuprofen lysine, were physicochemically compatible with caffeine citrate injection. Caffeine base injection was physicochemically compatible with all 43 test drugs tested.

**Supplementary Information:**

The online version contains supplementary material available at 10.1007/s00228-024-03678-6.

## Introduction

Caffeine is a respiratory stimulant used to treat apnoea of prematurity in neonates [1, 2]. The benefits of caffeine include a reduction in both the frequency of apnoea events and the requirement for mechanical ventilation in premature neonates [3–6]. Caffeine is also known to offer advantages over other medications used for apnoea (e.g. theophylline), including fewer serum concentration measurements (due to wider therapeutic index) and less frequent dosing (due to long elimination half-life) [7, 8].

In accordance with international treatment guidelines [9], the intravenous (IV) dosage regimen for caffeine (expressed as caffeine base) in neonates comprises a loading dose of 20 mg/kg (once only) and a maintenance dose of 5 to 7.5 mg/kg once daily (maximum 10 mg/kg/day) commencing 24 h after the loading dose. For typical loading doses, a caffeine concentration of 10 mg/mL (undiluted product) is administered by IV infusion over 30 min and for the maintenance dose, 5 mg/mL caffeine injection is infused over 10 min [10].

In neonatal intensive care unit (NICU) settings, multiple IV medications are often co-administered at high concentrations and low flow rates via Y-site (three-way) connectors [11, 12]. As these drugs are mixed in the IV tubing, physicochemical compatibility of co-administered drugs is an important consideration to avoid adverse clinical outcomes [12–14].

Compatibility information for caffeine citrate is mostly related to visually observable physical changes [15, 16]. Chemical compatibility for caffeine citrate is limited to very few drugs, reported in compendia from the manufacturer’s product information, including dopamine, fentanyl, heparin and calcium gluconate [17]. Stability data for caffeine base injection with parenteral nutrition (PN) solutions, IV fluids and admixtures have been reported [18]; however, there is a paucity of comprehensive physicochemical compatibility studies of caffeine base injection with other IV drugs. Caffeine base injection (10 mg/mL) is generally not commercially available, and this product is typically prepared by pharmaceutical compounding facilities as an isotonic formulation with a pH similar to caffeine citrate injection.

Our objective was to investigate the physicochemical compatibility of caffeine citrate and caffeine base injection with a range of NICU drugs, at higher-end, clinically relevant concentrations and with selected 2-in-1 PN solutions.

## Materials and methods

Caffeine (C_8_H_10_N_4_O_2_; MW 194.2; certified reference material) was purchased from Sigma-Aldrich Chemicals, St Louis, MO, USA. High-performance liquid chromatography (HPLC) grade acetonitrile was from Fisher Scientific, Fair Lawn, NJ, USA. All other laboratory chemicals were of analytical grade.

Caffeine citrate injection (20 mg/mL; equivalent to 10 mg/mL of caffeine base; Phebra Pty Ltd, Australia) and caffeine base injection (10 mg/mL; Perth Children’s Hospital, Australia) were tested against 43 secondary drugs and six 2-in-1 PN solutions, all of clinical grade (see Online Resource 1 for the list of drug manufacturers and composition of the PN solutions — Tables S1 and S2). Secondary drugs were prepared as per local NICU drug administration guidelines [10], using preferred diluents. Drug concentrations were based on the standard IV infusions for a patient weighing 2 kg.

The stability-indicating, HPLC assay method developed by Oliphant and colleagues [19] was modified and validated in accordance with the International Council for Harmonization (ICH) guidelines [20], for the determination of caffeine concentration in the present study (see Online Resource 1, Section 2, for details).

### Preparation of samples for physicochemical compatibility testing

Caffeine citrate and caffeine base injections were initially used undiluted (20 and 10 mg/mL concentrations respectively). Secondary test drugs and 2-in-1 PN solutions were prepared/diluted in accordance with the manufacturer’s instructions or standard neonatal clinical protocols [10]. Medications originally contained in glass ampoules and medications requiring reconstitution were filtered with a 0.22-µm syringe filter, before mixing (33 mm × 0.22 µm Polyethersulfone membrane, Millex-GP, Merk Millipore Ltd, Carrigtwohill, Co. Cork, Ireland).

A total of 43 drugs and 6 PN solutions were selected and endorsed by local clinical experts. These included drugs which were previously tested for physical compatibility, as compatible/incompatible controls.

Drug combinations were mixed at 1:1 volume ratio, to simulate Y-site administration, consistent with previously reported methods [15, 16, 21–24]. Drug preparation, mixing and testing were carried out at room temperature (22 °C).

The first stage of compatibility testing comprised a combination of caffeine citrate 20 mg/mL and caffeine base injection 10 mg/mL (separately) with the secondary drug at clinically relevant ‘high-end’ concentrations consistent with NICU protocols and expert advice. If incompatibility was detected, the secondary drug was then tested using caffeine citrate 10 mg/mL solution (diluted in water for injection), which is the recommended concentration for maintenance doses of caffeine [10]. If this combination also was incompatible, the next set of testing comprised caffeine citrate 20 mg/mL with the secondary drug at its ‘low-end’ concentration (if clinically applicable). Finally, the ‘lower-end’ caffeine concentration (caffeine citrate 10 mg/mL) was tested with the secondary drug ‘lower-end’ concentration, if previous results indicated this could be relevant.

Clear glass HPLC vials (2 mL) with impermeable screw cap lids were used for each binary combination of drugs/fluids and the respective control solutions. Initially, caffeine citrate and secondary drug combinations, and the control samples, were prepared as described below.

- Set 1 — Caffeine citrate injection solution (0.4 mL of 20 mg/mL) and secondary test drug solution (0.4 mL); *n* = 3.
- Set 2 — Caffeine citrate injection solution (0.4 mL of 20 mg/mL) diluted with 0.4 mL of the diluent of the secondary test drug (*n* = 3) as the reference control solution for the purpose of visual comparison and HPLC assay of caffeine concentration.
- Set 3 — The test drug solution (0.4 mL) diluted with 0.4 mL of water for injection (*n* = 3) for the purpose of visual comparison.

The same experimental procedure was followed for caffeine base injection (10 mg/mL) and conducted as a parallel experiment.

### Physical compatibility testing

All combinations were observed with an unaided eye against a black and white background for any change in colour, haze, precipitation and evolution of gas. The observations were carried out at time 0 (immediately after mixing), 5, 15, 60 and 120 min after mixing. Further, at time 0 and after 120 min, the samples were observed under a polarised light viewer (Apollo I Liquid Viewer with a LED light source and 1.7 × Magnifier, Adelphi Manufacturing Company Ltd, West Sussex, UK) for any precipitation or particulate matter.

Physical incompatibility was based on the visual appearance in comparison to control solutions (sets 2 and 3). Inconclusive observations were confirmed by a second independent observer and all physically incompatible combinations were photographed. If precipitation or particles were observed in the drug combination vials, an aliquot was examined under light microscopy (Leica MC190HD, 40 × magnification, Leica Microsystems Ltd, Heerbrugg, Switzerland).

### Chemical compatibility testing

If any physical incompatibility was observed (e.g. precipitate), the combinations were not subject to chemical compatibility testing, to avoid contamination of the HPLC system. Samples from sets 1 and 2 were analysed by HPLC after 2 h of observation. The ratio of the mean peak areas was determined, and the 95% confidence interval (CI) of the ratio was calculated using the confidence limits from a two-sided *t*-test (*α* = 0.05; SigmaPlot V.15; Inpixon GmbH, Düsseldorf, Germany). Consistent with previous studies, incompatibility of caffeine to drug combinations was defined as a ratio of the mean peak area outside the range of 90–110% [25–28].

## Results

Six of the 43 secondary drugs tested (aciclovir, amphotericin (liposomal), furosemide, hydrocortisone, ibuprofen and ibuprofen lysine) were physically incompatible with caffeine citrate undiluted injection, at their ‘high-end’ clinically relevant concentrations (Table 1). Two of the incompatible drugs were also tested at ‘low-end’ clinically relevant concentrations: hydrocortisone (1 mg/mL) was physicochemically compatible with caffeine citrate; however, furosemide (0.2 mg/mL) was physically incompatible (Table 1). All of the drugs which showed physical incompatibility with caffeine citrate undiluted injection were also physically incompatible with caffeine citrate 10 mg/mL solution (Table 2).

**Table 1:** Physicochemical compatibility of caffeine citrate 20 mg/mL (10 mg/mL caffeine base) with secondary drugs 2-in-1 parenteral nutrition solutions (see Table S2 for details)

**Secondary drug**	**Test concentration**	**Diluent**	**PC**	**CAF ratio**	**95% CI of ratio**
Aciclovir	5 mg/mL	D5W	I^a^	-	-
Alprostadil	20 mcg/mL	NS	C	99.9	98.1–101.7
Amoxicillin	100 mg/mL	WFI	C	99.9	99.1–100.8
Amphotericin (Fungizone)	100 mcg/mL	D5W	C	98.8	97.1–100.6
Amphotericin liposomal	2 mg/mL	D5W	I^b^	-	-
Ampicillin	100 mg/mL	WFI	C	99.8	98.9–100.7
Benzylpenicillin	100 mg/mL	WFI	C	100.6	99.4–101.7
Calcium gluconate	100 mg/mL	U	C	99.5	98.2–100.9
Cefotaxime	100 mg/mL	WFI	C	100.2	99.3–101.2
Ciprofloxacin*	2 mg/mL	U	C	100.2	99.5–100.9
Clonidine	2 mcg/mL	NS	C	99.6	98.2–101.0
Cloxacillin	100 mg/mL	WFI	C	100.1	98.2–101.9
Dobutamine	7.2 mg/mL	NS	C	100.1	99.3–100.9
Dobutamine	7.2 mg/mL	D5W	C	100.0	98.8–101.3
Dopamine	7.2 mg/mL	NS	C	100.0	99.4–100.6
Dopamine	7.2 mg/mL	D5W	C	100.6	99.3–101.8
Dexmedetomidine	1 mcg/mL	NS	C	99.7	98.4–101.1
Epinephrine	64 mcg/mL	D5W	C	100.1	99.5–100.7
Fentanyl	50 mcg/mL	U	C	99.8	98.7–101.0
Flucloxacillin	50 mg/mL	D5W	C	99.2	96.9–101.5
Fluconazole	2 mg/mL	U	C	99.5	98.6–100.5
Furosemide	1 mg/mL	D5W	I^a^	-	-
Furosemide	0.2 mg/mL	D5W	I^c^	-	-
Gentamicin	10 mg/mL	NS	C	99.6	99.1–100.1
Heparin	100 units/mL	NS	C	99.7	98.5–100.8
Hydrocortisone	10 mg/mL	NS	I^a^	-	-
Hydrocortisone	1 mg/mL	NS	C	99.6	97.5–101.7
Indometacin	200 mcg/mL	NS	C	99.5	98.7–100.3
Ibuprofen	5 mg/mL	NS	I^d^	-	-
Ibuprofen lysine	4 mg/mL	NS	I^d^	-	-
Insulin	0.2 units/mL	NS	C	99.7	98.8–100.6
Levetiracetam	5 mg/mL	NS	C	99.5	99.0–100.0
Linezolid	2 mg/mL	U	C	99.9	98.8–100.9
Meropenem	50 mg/mL	NS	C	98.9	97.1–100.8
Metronidazole	5 mg/mL	U	C	99.9	98.9–101.0
Midazolam	1 mg/mL	U	C	99.5	97.8–101.3
Milrinone	400 mcg/mL	D5W	C	100.2	99.7–100.8
Morphine hydrochloride	200 mcg/mL	D5W	C	99.8	98.4–101.2
Morphine sulfate	200 mcg/mL	D5W	C	100.6	99.5–101.8
Norepinephrine	64 mcg/mL	D5W	C	100.3	99.7–101.0
Paracetamol	10 mg/mL	U	C	100.2	99.3–101.1
Phenobarbitone	20 mg/mL	WFI	C	99.8	99.0–100.6
Piperacillin/tazobactam	200 mg/mL	WFI	C	99.8	98.8–100.7
Rifampicin	6 mg/mL	NS	C	100.4	98.6–102.1
Sodium bicarbonate	4.2% w/v	D5W	C	99.4	98.9–99.8
Vancomycin	10 mg/mL	D5W	C	99.3	97.8–100.8
Vecuronium	1 mg/mL	WFI	C	99.7	99.4–100.1
Parenteral nutrition PN 1	-	-	C	99.2	97.7–100.8
Parenteral nutrition PN 2	-	-	C	100.5	99.1–102.0
Parenteral nutrition PN 3	-	-	C	99.6	98.9–100.2
Parenteral nutrition PN 4	-	-	C	99.9	98.9–101.0
Parenteral nutrition PN 5	-	-	C	100.5	99.4–101.6
Parenteral nutrition PN 6	-	-	C	99.7	98.8–100.6

*PC* physical compatibility, *CAF* caffeine, *C* compatible, *I* incompatible, *D5W* glucose 5%, *WFI* water for injection, *NS* normal saline/ 0.9% sodium chloride, *U* undiluted

*Ciprofloxacin was also tested at 4 h to obtain a caffeine ratio of 99.6% and a 95% CI of ratio 98.1–101.1%

^a^A white precipitate appeared 10–15 min after mixing

^b^A higher opacity observed in the combination samples in comparison to controls

^c^Particles observed under polarised light after 30 min of mixing

^d^A milky turbidity appeared immediately after mixing

**Table 2:** Physicochemical compatibility of caffeine citrate 10 mg/mL (5 mg/mL caffeine base) with secondary drugs

**Secondary drug**	**Test concentration**	**Diluent**	**PC**
Aciclovir	5 mg/mL	D5W	I^a^
Amphotericin liposomal	2 mg/mL	D5W	I^b^
Furosemide	1 mg/mL	D5W	I^a^
Furosemide	0.2 mg/mL	D5W	I^c^
Hydrocortisone	10 mg/mL	NS	I^a^
Ibuprofen	5 mg/mL	NS	I^d^
Ibuprofen lysine	4 mg/mL	NS	I^d^

*PC* physical compatibility, *I* incompatible, *D5W* glucose 5% w/v, *NS* normal saline (sodium chloride 0.9% w/v)

^a^A white precipitate appeared 10–15 min after mixing

^b^A higher opacity observed in the combination samples in comparison to controls

^c^Particles observed under polarised light after 30 min of mixing

^d^A milky turbidity appeared immediately after mixing

Most of the physical incompatibilities were visible to the unaided eye (Online Resource 1 for photographs, Figs. S3–S8), except the combinations with furosemide 0.2 mg/mL, which required observation under polarised light. As amphotericin (liposomal) was originally a pale-yellow hazy mixture, the incompatibility observed was an increase in the opacity in comparison to the control mixtures (Online Resource1; Fig. S4).

Further investigation of the incompatibility findings was conducted by mixing the six secondary drugs (separately, as described in the “Preparation of samples for physicochemical compatibility testing” section) with citrate buffer pH 4.5 (citric acid monohydrate 5 mg/mL and sodium citrate dihydrate 8.3 mg/mL in water). The same physical incompatibility characteristics (precipitation/haze) were observed with all six secondary drugs (Online Resource 1; Fig. S9), therefore indicating the citrate buffer was the cause of the incompatibility with caffeine citrate injection.

In contrast to the caffeine citrate data, all 43 secondary drugs and 6 PN solutions tested were physicochemically compatible with caffeine base injection (Table 3).

**Table 3:** Physicochemical compatibility of caffeine base injection 10 mg/mL with secondary drugs 2-in-1 parenteral nutrition solutions (see Table S2 for details)

**Secondary drug**	**Test concentration**	**Diluent**	**PC**	**CAF ratio**	**95% CI of ratio**
Aciclovir	5 mg/mL	D5W	C	99.9	98.4–101.4
Alprostadil	20 mcg/mL	NS	C	99.4	97.8–101.1
Amoxicillin	100 mg/mL	WFI	C	100.3	99.6–100.9
Amphotericin (Fungizone)	100 mcg/mL	D5W	C	101.2	98.9–103.5
Amphotericin liposomal	2 mg/mL	D5W	C	100.2	98.8–101.6
Ampicillin	100 mg/mL	WFI	C	100.3	98.5–102.1
Benzylpenicillin	100 mg/mL	WFI	C	100.2	98.7–101.6
Calcium gluconate	100 mg/mL	U	C	100.3	99.1–101.5
Cefotaxime	100 mg/mL	WFI	C	99.4	96.9–101.8
Ciprofloxacin	2 mg/mL	U	C	99.7	99.1–100.3
Clonidine	2 mcg/mL	NS	C	99.6	98.2–101.1
Cloxacillin	100 mg/mL	WFI	C	100.7	99.6–101.7
Dobutamine	7.2 mg/mL	NS	C	100.8	98.4–103.2
Dobutamine	7.2 mg/mL	D5W	C	100.0	99.2–100.8
Dopamine	7.2 mg/mL	NS	C	100.6	99.6–101.5
Dopamine	7.2 mg/mL	D5W	C	100.7	99.3–102.1
Dexmedetomidine	1 mcg/mL	NS	C	100.9	99.8–102.0
Epinephrine	64 mcg/mL	D5W	C	99.9	99.3–100.4
Fentanyl	50 mcg/mL	U	C	100.0	98.7–101.4
Flucloxacillin	50 mg/mL	D5W	C	99.8	98.4–101.3
Fluconazole	2 mg/mL	U	C	99.6	98.6–100.6
Furosemide	1 mg/mL	D5W	C	100.4	99.2–101.7
Gentamicin	10 mg/mL	NS	C	99.8	98.9–100.7
Heparin	100 units/mL	NS	C	100.1	99.4–100.8
Hydrocortisone	10 mg/mL	NS	C	100.6	99.2–101.9
Indometacin	200 mcg/mL	NS	C	100.4	99.6–101.2
Ibuprofen	5 mg/mL	NS	C	99.8	98.5–101.2
Ibuprofen lysine	4 mg/mL	NS	C	99.9	98.7–101.0
Insulin	0.2 units/mL	NS	C	101.2	98.3–104.2
Levetiracetam	5 mg/mL	NS	C	101.1	100.2–102.0
Linezolid	2 mg/mL	U	C	100.4	97.8–103.0
Meropenem	50 mg/mL	NS	C	99.8	98.8–100.7
Metronidazole	5 mg/mL	U	C	100.4	99.5–101.3
Midazolam	1 mg/mL	U	C	99.5	98.8–100.2
Milrinone	400 mcg/mL	D5W	C	99.4	98.2–100.5
Morphine hydrochloride	200 mcg/mL	D5W	C	99.9	98.8–101.0
Morphine sulfate	200 mcg/mL	D5W	C	99.6	98.3–101.0
Norepinephrine	64 mcg/mL	D5W	C	99.7	99.1–100.4
Paracetamol	10 mg/mL	U	C	100.0	99.6–100.5
Phenobarbitone	20 mg/mL	WFI	C	100.5	98.9–102.0
Piperacillin/tazobactam	200 mg/mL	WFI	C	100.0	99.0–101.0
Rifampicin	6 mg/mL	NS	C	101.4	99.1–103.6
Sodium bicarbonate	4.2% w/v	D5W	C	99.3	98.4–100.3
Vancomycin	10 mg/mL	D5W	C	99.9	98.1–101.6
Vecuronium	1 mg/mL	WFI	C	100.3	98.6–102.0
Parenteral nutrition PN 1	-	-	C	100.1	99.1–101.1
Parenteral nutrition PN 2	-	-	C	100.3	98.2–102.4
Parenteral nutrition PN 3	-	-	C	99.3	98.0–100.7
Parenteral nutrition PN 4	-	-	C	99.7	98.2–101.2
Parenteral nutrition PN 5	-	-	C	100.6	99.3–101.9
Parenteral nutrition PN 6	-	-	C	99.3	97.6–101.1

*PC* physical compatibility, *CAF* caffeine, *C* compatible, *D5W* glucose 5% w/v, *WFI* water for injection, *NS* normal saline (sodium chloride 0.9% w/v), *U* undiluted

To complement the above results, the osmolality of the caffeine citrate 20 mg/mL and caffeine base 10 mg/mL injections was tested and found to be 142 and 269 mOsm/kg, respectively (Osmomat 030 Cryoscopic Osmometer; Gonotec GmbH, Berlin, Germany). By comparison, a recent report indicated that caffeine citrate 20 mg/mL oral solution had an osmolality of 150 mOsm/kg [29].

## Discussion

The present study has shown that 37 IV drugs tested in a simulated Y-site study design at ‘high-end’, clinically relevant concentrations for NICU settings were physically and chemically compatible with caffeine citrate 20 mg/mL injection (Table 1). The apparent cause of the incompatibility of caffeine citrate injection with aciclovir, amphotericin (liposomal), furosemide, hydrocortisone, ibuprofen and ibuprofen lysine injections was found to be the citrate buffer component. By comparison, all 43 drugs were compatible with caffeine base 10 mg/mL injection (Table 3). Caffeine citrate and base injections were also compatible with six 2-in-1 parenteral nutrition solutions.

Although physical compatibility information for caffeine citrate with a range of IV drugs has been reported, a modest compilation of chemical compatibility data from manufacturers’ information (for dopamine, fentanyl, heparin and calcium gluconate) is available in contemporary guidelines [17]. Consistent with these data, our study demonstrated physicochemical compatibility of caffeine citrate injection with calcium gluconate, dopamine, fentanyl and heparin, albeit at different concentrations and/or experimental conditions. For example, a mixture of caffeine citrate 20 mg/mL and calcium gluconate 100 mg/mL was previously found to be physically compatible for 4 [16] and 24 h [17] at room temperature, and chemically stable for 24 h at room temperature [17]. These findings provide useful confirmation of our results that caffeine citrate and calcium gluconate injections were physicochemically compatible for 2 h at room temperature.

Heparin has previously been investigated at 1 unit/mL (in glucose 5% w/v; D5W), 10 units/mL and 1000 units/mL in combination with caffeine citrate and shown to be physically compatible [15–17]. The present study complements these reports by demonstrating that heparin 100 units/mL was physicochemically compatible with caffeine citrate, for 2 h at room temperature (Table 1).

Fentanyl 10 mcg/mL (in D5W) was reported to be compatible and stable with caffeine citrate for 24 h at room temperature [17], and two studies have confirmed that fentanyl 50 mcg/mL was physically compatible for 4 h at room temperature [15, 16]. Furthermore, meropenem 50 mg/mL was recently found to be physically compatible with caffeine citrate injection for 4 h [30]. Hence, these results also are complemented by the present study, whereby fentanyl 50 mcg/mL and meropenem 50 mg/mL (separately) were found to be physically and chemically compatible with caffeine citrate injection (Table 1).

The present study also provides evidence of incompatibility between caffeine citrate injection (10 mg/mL and 20 mg/mL) and both ibuprofen (5 mg/mL) and ibuprofen lysine (4 mg/mL), the combinations of which resulted in turbidity immediately after mixing (Figs. S7 and S8). Although ibuprofen has not been studied previously for physicochemical compatibility, ibuprofen lysine 20 mg/mL was reported to be physically incompatible due to milky white precipitation upon mixing [31].

A range of inconsistent caffeine citrate compatibility data have been reported, some of which may be concentration-dependent or related to the experimental procedures (e.g. duration of admixture or physical methods used to determine compatibility), or the composition of the IV drug formulation [16]. For example, dopamine 0.6 mg/mL (in D5W) was reported to be compatible and stable with caffeine citrate for 24 h at room temperature [17], and a higher concentration (80 mg/mL) was found to be visually compatible for 4 h at 25 °C [15]. By contrast, Audet and colleagues [16] reported that dopamine 3.2 mg/mL was physically incompatible with caffeine citrate, due to a ‘yellowish tint’ colour change immediately after mixing. However, in the present study, dopamine 7.2 mg/mL (in both D5W and 0.9% sodium chloride; NS) was physically and chemically compatible with caffeine citrate for 2 h after mixing (Table 1). Furthermore, for direct comparison with the previous report [16], we investigated the combinations of caffeine citrate 20 mg/mL injection with dopamine 3.2 and 1.2 mg/mL (in NS) and found no evidence of physicochemical incompatibility (physically compatible with no observed colour change and caffeine ratios of 99.4% and 99.1%, respectively).

Conflicting data regarding the compatibility of caffeine citrate with furosemide 10 mg/mL and aciclovir 50 mg/mL (separately) also have been reported, with one study finding the combinations were physically compatible [16], and an earlier study indicating they were physically incompatible, due to immediate precipitation [15]. By comparison, the present study has shown that lower, clinically relevant concentrations of these drugs (furosemide 1 and 0.2 mg/mL, and aciclovir 5 mg/mL) were physically incompatible with caffeine citrate, as the combinations produced a white precipitate within 15 min of mixing (Table 1 and Figs. S3 and S5). These results may indicate concentration-dependent physical incompatibility for mixtures of caffeine citrate and furosemide or aciclovir, which could be evaluated in clinical settings, based on the presence/absence of a visible white precipitate.

In regard to amphotericin (liposomal) and hydrocortisone, at concentrations of 4 mg/mL and 250 mg/mL respectively, Audet et al. [16] found these two drugs were physically compatible with caffeine citrate for 4 h at room temperature. By contrast, results in the present study showed that amphotericin (liposomal) and hydrocortisone, at lower clinically relevant NICU concentrations (2 mg/mL and 10 mg/mL respectively), were physically incompatible with caffeine citrate at 10 mg/mL and 20 mg/mL (Table 1 and Figs. S4 and S6). However, hydrocortisone at a concentration of only 1 mg/mL was physicochemically compatible with caffeine citrate 20 mg/mL (Table 1). This finding suggests the lower hydrocortisone IV infusion concentration (1 mg/mL) used in NICU settings may be safely co-administered with caffeine citrate through Y-sites, where required.

Audet et al. [16] also reported that midazolam 5 mg/mL was physically incompatible with caffeine citrate, due to the formation of a white precipitate at the time of mixing; however, our study showed that a lower concentration (1 mg/mL) was physicochemically compatible with caffeine citrate (Table 1).

Further contradictory studies regarding vancomycin 50 mg/mL or dobutamine 12.5 mg/mL mixed (separately) with caffeine citrate have reported the combinations to be physically compatible [15] and physically incompatible [16], resulting in white precipitate and colour change, respectively, at the time of mixing in the latter study. By comparison, we found that vancomycin and dobutamine, at the lower concentrations of 10 mg/mL and 7.2 mg/mL, respectively (in both D5W and NS), were physicochemically compatible with caffeine citrate 20 mg/mL (Table 1).

One directly conflicting result from the present study relates to the recent report that ciprofloxacin 2 mg/mL was physically incompatible with caffeine citrate 20 mg/mL due to crystal formation at 4 h after mixing [16]. By contrast, our data indicate the combination is physicochemically compatible for 2 h at the same concentrations. Hence, to clarify this discrepancy and formally compare our study with the previous report [16], we retested the combination after 4 h of mixing and confirmed its physicochemical compatibility in our laboratory, with no physical evidence of precipitate or crystal formation and a caffeine concentration ratio (by HPLC) of 99.6% (Table 1). As outlined above, similar inexplicable discrepancies are evident in specific studies [16] and compendia [17], and may require prudent clinical judgement to avoid adverse clinical outcomes.

Compared to the studies of caffeine citrate compatibility, there are no previous comprehensive physical or chemical compatibility studies of caffeine base injection with other IV drugs. However, the stability of caffeine base in a range of sodium chloride, potassium chloride and glucose IV solutions and PN fluids for up to 24 h has been reported [18]. The present investigation has shown that caffeine base injection was physicochemically compatible with all 43 secondary drugs and the six PN solutions tested (Table 3). Hence, in the absence of commercial preparations, a locally prepared caffeine base injection may be a useful alternative to caffeine citrate injection for Y-site co-administration with otherwise incompatible IV drugs.

One potential limitation of the present study was the well-established, fixed 1:1 mixing ratio of the two components for simulated Y-site compatibility studies [16, 21, 26, 32]. Recent reports have included other ratios (e.g. 1:4 or 1:10) to simulate extremes of high/low infusion rates of the individual components [27, 33–35]; however, in the NICU setting, the range of drug concentrations may be a more significant variable than the IV infusion rates. Nevertheless, contemporary IV compatibility study designs could include a balanced range of clinically relevant concentrations and mixing ratios, as appropriate. A further consideration in our study was the 2-h mixing duration, which is based on the typical contact time of two components in neonatal infusions (via Y-site mixing) being up to 1 h [36, 37], but accounts for potentially slower infusion rates that may occur in NICU settings [16]. Finally, some recent physical and physicochemical compatibility investigations have included turbidity and/or pH tests as part of the suite of physical tests [26, 28, 38]; however, due to resource implications for these tests, including the large sample volumes (typically > 10 mL), turbidity and pH were not evaluated in the present study. Furthermore, recent reports have noted the intrinsic value, interpretation and specification limits of some physical compatibility tests are unclear or inconsistent [26, 28, 38, 39]. Hence, based on the range of well-accepted physical tests and validated HPLC assay for determination of chemical compatibility, we conclude the present study provides sufficiently robust evidence of physicochemical compatibility (or otherwise) for caffeine citrate and caffeine base injections in the context of simulated Y-site co-administration in NICU settings.

## Conclusion

Most secondary test drugs and 2-in-1 PN solutions investigated in the present study, except aciclovir, amphotericin (liposomal), furosemide, hydrocortisone, ibuprofen and ibuprofen lysine, were physicochemically compatible with caffeine citrate injection (20 mg/mL). By comparison, caffeine base injection (10 mg/mL) was physicochemically compatible with all 43 test drugs and six PN solutions tested.

### Supplementary Information

Below is the link to the electronic supplementary material.

Supplementary file1 (PDF 594 KB)

## Data Availability

All data supporting the findings of this study are available within the paper or its Supplementary Information or are available on reasonable request to the authors.
