# Supplementary material for: Physicochemical compatibility of caffeine citrate and caffeine base injections with parenteral medications used in neonatal intensive care settings
Source: Eur J Clin Pharmacol. 2024 Mar 28;80(7):1079–87. doi: 10.1007/s00228-024-03678-6 (PMC11156738; doi:10.1007/s00228-024-03678-6)
Supplement: Supplementary file 1 — Supplementary file1 (PDF 594 KB) [file 228_2024_3678_MOESM1_ESM.pdf]

Supplementary information

Physicochemical compatibility of caffeine citrate and caffeine base injections with parenteral medications used in neonatal intensive care settings

D. Thisuri N. De Silva,[1] Michael Petrovski,[2] Tobias Strunk,[3,4,5] Nabeelah Mukadam,[6] Madhu Page-Sharp,[1] Brioni R. Moore,[1,3,4,7] Kevin T. Batty[1,7]*

[1] Curtin Medical School, Curtin University, Bentley, Western Australia

[2] Pharmacy Department, Sir Charles Gardiner Hospital, North Metropolitan Health Service, Nedlands, Western Australia

[3] Medical School, The University of Western Australia, Crawley, Western Australia

[4] Wesfarmers Centre for Vaccines and Infectious Diseases, Telethon Kids Institute, Nedlands, Western Australia

[5] Neonatal Directorate, King Edward Memorial Hospital, Child and Adolescent Health Service, Subiaco, Western Australia

[6] Pharmacy Department, King Edward Memorial Hospital, Women and Newborn Health Service, Subiaco, Western Australia

[7] Curtin Health Innovation Research Institute, Curtin University, Bentley, Western Australia

* Corresponding author: Professor Kevin Batty, Curtin Medical School, Curtin University, GPO Box U1987, Perth, Western Australia, 6845. Telephone: (61-8) 9266 2535; Email: Kevin.Batty@curtin.edu.au

Section 1: Manufacturers/suppliers of injectable products (Table S1) and composition of the 2-in-1 parenteral nutrition solutions (Table S2)

Table S1. Manufacturers/ suppliers of injectable products used for compatibility studies.

Injectable drug	Manufacturer/ supplier
Aciclovir	Pfizer Australia Pty Ltd, Sydney, NSW, Australia
Alprostadil	Pfizer Australia Pty Ltd, Sydney, NSW, Australia
Amoxicillin	Juno Pharmaceuticals Pty Ltd, Cremorne, VIC, Australia
Amphotericin B - Fungizone	XGen Pharmaceuticals DJB, NY 14814, United States
Amphotericin B - Liposomal	Gilead Sciences Pty Ltd, St Kilda Road, Melbourne, VIC, Australia
Ampicillin	Juno Pharmaceuticals Pty Ltd, Cremorne, VIC, Australia
Benzylpenicillin	Seqirus (Australia) Pty Ltd, Melbourne, VIC, Australia
Caffeine base*	Perth Childrens' Hospital, Nedlands, WA, Australia
Caffeine citrate	Phebra Pty Ltd, Lane Cove West, NSW, Australia
Calcium gluconate	Phebra Pty Ltd, Lane Cove West, NSW, Australia
Cefotaxime	Pfizer Australia Pty Ltd, Sydney, NSW, Australia
Ciprofloxacin	Aspen Pharmacare Australia Pty Ltd, Leonards, NSW, Australia
Clonidine	Medicianz Healthcare Pty Ltd, Melbourne, VIC, Australia
Cloxacillin	SteriMax Inc, Oakville, ON L6H6R4, Canada
Dexmedetomidine	Accord Healthcare Pty Ltd, Melbourne, VIC, Australia
Dobutamine	Pfizer Australia Pty Ltd, Sydney, NSW, Australia
Dopamine	Juno Pharmaceuticals Pty Ltd, Cremorne, VIC, Australia
Epinephrine	Aspen Pharmacare Australia Pty Ltd, Leonards, NSW, Australia
Fentanyl citrate	Piramal Critical Care Pty Ltd, Chatswood, NSW, Australia
Flucloxacillin	Juno Pharmaceuticals Pty Ltd, Cremorne, VIC, Australia
Fluconazole	Pfizer Australia Pty Ltd, Sydney, NSW, Australia
Furosemide	Baxter Health care Pty Ltd, Old Toongabbie, NSW, Australia
Gentamicin	Pfizer Australia Pty Ltd, Sydney, NSW, Australia
Heparin	Pfizer Australia Pty Ltd, Sydney, NSW, Australia
Hydrocortisone	Pfizer Australia Pty Ltd, Sydney, NSW, Australia
Ibuprofen	Seqirus (Australia) Pty Ltd, Melbourne, VIC, Australia
Ibuprofen lysine	Prasco Laboratories, Commerce Ct, Mason, United States
Indometacin	Promedica SRL, Via Palermo, Parma, Italy
Insulin	Novo Nordisk Pharmaceuticals Pty Ltd, Baulkham Hills, NSW, Australia
Levetiracetam	Apotex Pty Ltd, Macquarie Park, NSW, Australia
Linezolid	Fresenius Kabi Australia Pty Ltd, Mount Kuring-gai, NSW, Australia
Meropenem	Sun Pharma ANZ Pty Ltd, Macquarie Park, NSW, Australia
Metronidazole	Juno Pharmaceuticals Pty Ltd, Cremorne, VIC, Australia
Midazolam	Pharmaco (Australia) Ltd, Gordon, NSW, Australia
Milrinone	Generic Health Pty Ltd, Box Hill, VIC, Australia
Morphine hydrochloride	Juno Pharmaceuticals Pty Ltd, Cremorne, VIC, Australia
Morphine sulfate	Pfizer Australia Pty Ltd, Sydney, NSW, Australia
Norepinephrine	Juno Pharmaceuticals Pty Ltd, Cremorne, VIC, Australia
Paracetamol	B.Braun Australia Pty Ltd, Bella Vista, NSW, Australia
Phenobarbitone	Aspen Pharmacare Australia Pty Ltd, Leonards, NSW, Australia
Piperacillin/tazobactam	Sandoz Pty Ltd, Macquarie Perk, NSW, Australia

Injectable drug	Manufacturer/ supplier
Rifampicin	Sanofi-Aventis Australia Pty Ltd, Macquarie Park, NSW, Australia
Sodium bicarbonate	Phebra Pty Ltd, Lane Cove West, NSW, Australia
Vancomycin	Alphapharm Pty Ltd, Carole Park, QLD, Australia
Vecuronium	Sun Pharma ANZ Pty Ltd, Macquarie Park, NSW, Australia

*Caffeine base 10 mg/mL injection comprises caffeine, sodium chloride, hydrochloride acid and Water for Injection; the injection is isotonic and has a pH approximately 4.2 (AUSPMAN/ Perth Children's Hospital)

Table S2. Composition of the 2-in-1 parenteral nutrition solutions, manufactured at King Edward Memorial Hospital

	PN 1 Preterm A	PN 2 Preterm B	PN 3 Term	PN 4 Custom 1	PN 5 Custom 2	PN 6 Custom 3
Amino acid g/100mL	2.7	2.7	2.3	0.5	3.5	2.3
Glucose, g/100mL	5	8	12	2	14	8
Sodium, mmol/100mL	4	4	4	4	4	4
Potassium, mmol/100mL	2	2	2	2	2	2
Calcium, mmol/100mL	1.5	1.5	0.9	1.5	1.5	1.5
Phosphate, mmol/100mL	1.5	1.5	0.9	1.5	1.5	1.5
Magnesium, mmol/100mL	0.25	0.25	0.25	0.25	0.25	0.25
Acetate, mmol/100mL	2	2	2.56	1.79	2.08	1.96
Chloride, mmol/100mL	2.01	2.01	2.57	1.8	2.08	1.97
Trace elements, mL/100mL	-	-	0.74	0.74	0.74	0.74
Heparin, units/100mL	50	50	50	50	50	50

Section 2. High performance liquid chromatography (HPLC) assay method and validation

An Apollo C_{18} HPLC column (150 × 4.6 mm, 5 µm; Hichrom Ltd, Berkshire, England) was used for chromatographic separation. The isocratic mobile phase comprised 85% water and 15% acetonitrile (ACN) v/v at a flow rate of 0.9 mL/min. The column oven temperature was 30°C, the injection volume was 1 µL and the UV detection wavelength was 273 nm.

The Agilent 1200 series HPLC system comprised a binary pump with degasser, auto-sampler, thermostated column oven and a dual wavelength UV detector (Agilent Technology, Waldbronn, Germany). Chemstation software (vRev. B.03.01.SR1; Agilent Technology) was used to acquire and process data.

The caffeine retention time was approximately 5.8 minutes (Figure S1).

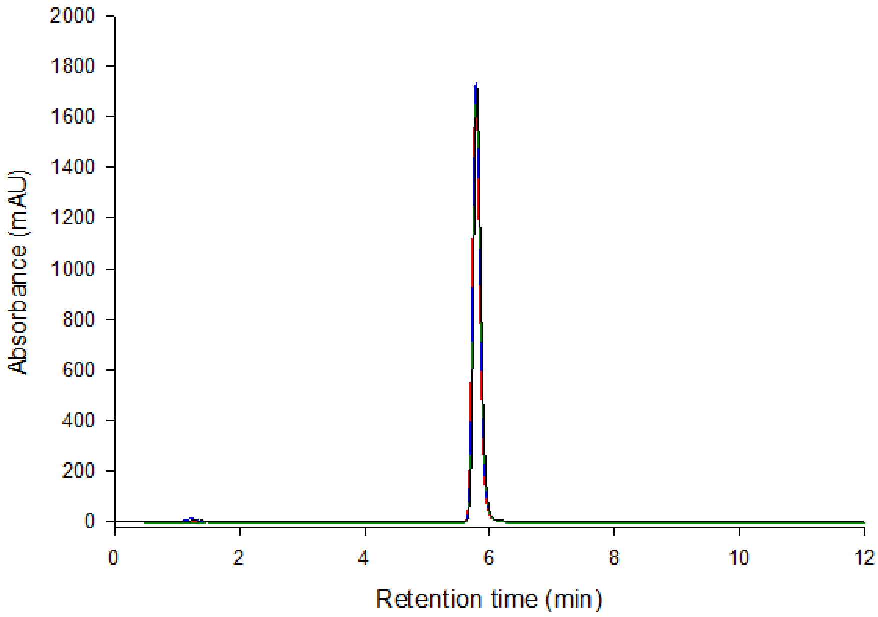

Figure S1 – Chromatograms of caffeine standard 5 mg/mL (—), caffeine citrate 10 mg/mL (—), caffeine base injection 5 mg/mL (—), a mixture of caffeine standard, caffeine citrate and caffeine base injection in aqueous solution (—); caffeine retention time approximately 5.8 minutes

Linearity and range

To establish linearity and range for the HPLC assay, a calibration curve was constructed using caffeine (base) solutions at concentrations of 1, 2, 3, 4, and 5 mg/mL (n = 3). Calibration curve and analyte concentration data were analysed using Microsoft Excel (Version 2309 Build 16.0.16827.20166). The limit of detection (LOD) was estimated using the formula LOD = 3.3 σ/S and the lower limit of quantification (LLOQ) was estimated using the formula LLOQ = 10 σ/S, where σ was the residual standard deviation of the regression line and S was the slope of the calibration curve. LLOQ was confirmed by precision data.

The assay was linear for caffeine in aqueous solution (n = 3) within the concentration range 1 to 5 mg/mL ($r^2 > 0.999$) (Figure S2). The LOD and LLOQ for caffeine were 0.07 and 0.2 mg/mL respectively.

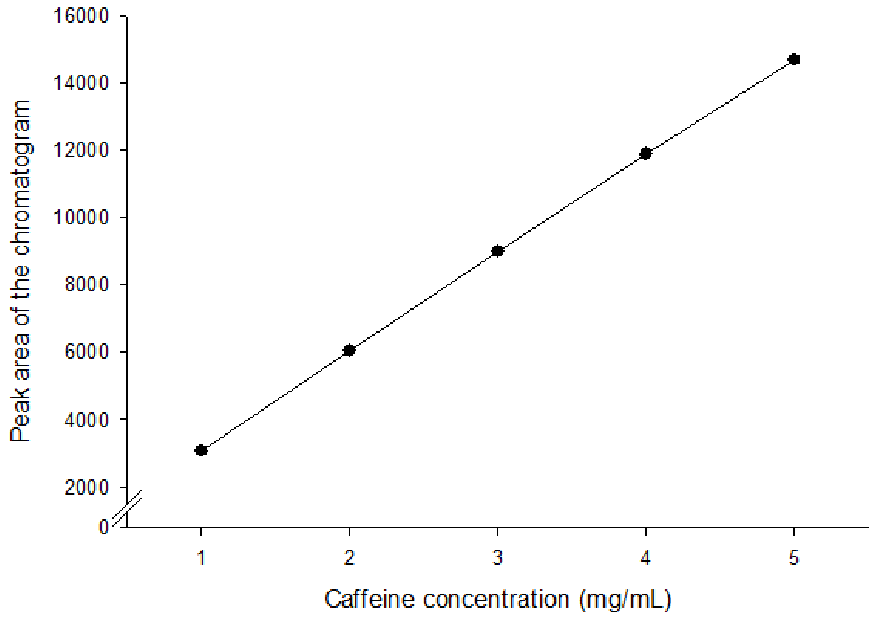

Figure S2 – Linearity curve for caffeine solution in aqueous solution within the concentration range 1 – 5 mg/mL (n = 3); correlation coefficient (r^2) > 0.999; regression equation Y = 2907x + 217.13

Accuracy and precision

Accuracy and precision of the HPLC assay was evaluated at caffeine concentrations of 5, 3, 1 and 0.2 (LLOQ) mg/mL (n = 5) using the caffeine reference standard, and both the commercial caffeine citrate injection and caffeine base injections diluted with water. The concentrations of the two series were compared (expressed as a fraction of the nominal concentration). Intra-assay and inter-assay precision were determined by calculating percentage Relative Standard Deviation (%RSD) for the same caffeine concentrations.

The HPLC method was accurate and precise according to the standard definitions, with accuracy between 98 and 101 % for all concentrations, and precision (as % RSD) ≤ 1% for inter- and intra assay samples (Table S3).

Table S3. Accuracy, intra-assay, and inter-assay precision data for selected caffeine concentrations (caffeine citrate and caffeine base injection)

Concentration (mg/mL); n = 5	Caffeine concentration as a % of nominal concentration (Mean ± SD, n=5)		Intra assay-precision (% RSD)		Inter-assay precision (% RSD pooled)	
	Caffeine citrate	Caffeine base	Caffeine citrate	Caffeine base	Caffeine citrate	Caffeine base
LLOQ	99.3 ± 0.7	99.2 ± 0.1	0.5	0.4	0.4	0.5
1	98.0 ± 0.8	101.1 ± 1.0	0.5	1.0	0.7	0.6
3	98.0 ± 0.8	100.0 ± 1.5	0.4	0.3	0.4	0.4
5	98.3 ± 0.4	99.4 ± 0.6	0.4	0.4	0.2	0.3

Robustness of the adopted HPLC method

The robustness of an analytical procedure is a measure of its capacity to remain unaffected by small, but deliberate variations in method parameters and provides an indication of its reliability during normal usage. To evaluate robustness, caffeine 5 mg/mL (as caffeine base) samples (from caffeine standard, caffeine citrate commercial injection and caffeine base injection; n = 5) were tested using the modified method. Changes with respect to standard method parameters included flow rate (1.0 mL/min) and mobile phase composition (water: ACN 80:20). The accuracy of the modified methods was compared with the standard method. The percentage concentrations of caffeine in robustness testing experiment revealed that the method was robust despite deliberate minute changes in method parameters (Table S4).

Table S4. Robustness test results for deliberate changes in method parameters

Parameters	Conditions	Caffeine concentration as a % of nominal concentration, in caffeine citrate injection (Mean ± SD, n=5)	Caffeine concentration as a % of nominal concentration, in caffeine base injection (Mean ± SD, n=5)
Flow rate	0.9 mL/min	98.3 ± 0.4	99.4 ± 0.6
	1.0 mL/min	98.9 ± 0.7	99.7 ± 0.4
Mobile phase composition (water: ACN)	85:15	98.3 ± 0.4	99.4 ± 0.6
	80:20	99.2 ± 0.3	99.6 ± 0.6

Section 3. Photographs and corresponding photomicrographs of physical incompatibilities of caffeine citrate (20 mg/mL) with aciclovir, amphotericin (liposomal), furosemide, hydrocortisone, ibuprofen and ibuprofen lysine (Figure S3 – 8); Citrate buffer incompatibilities with drugs (Figure S9)

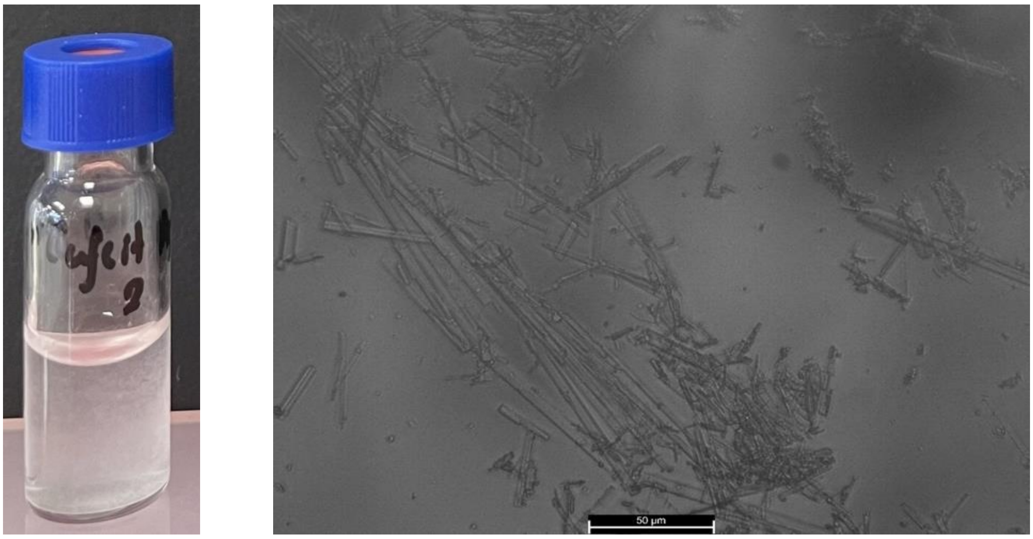

Figure S3 – Photograph (left) and corresponding photomicrograph (right) (Leica MC190HD, objective x40) of the white precipitate observed in the combination of caffeine citrate (20 mg/mL) and aciclovir (5 mg/mL)

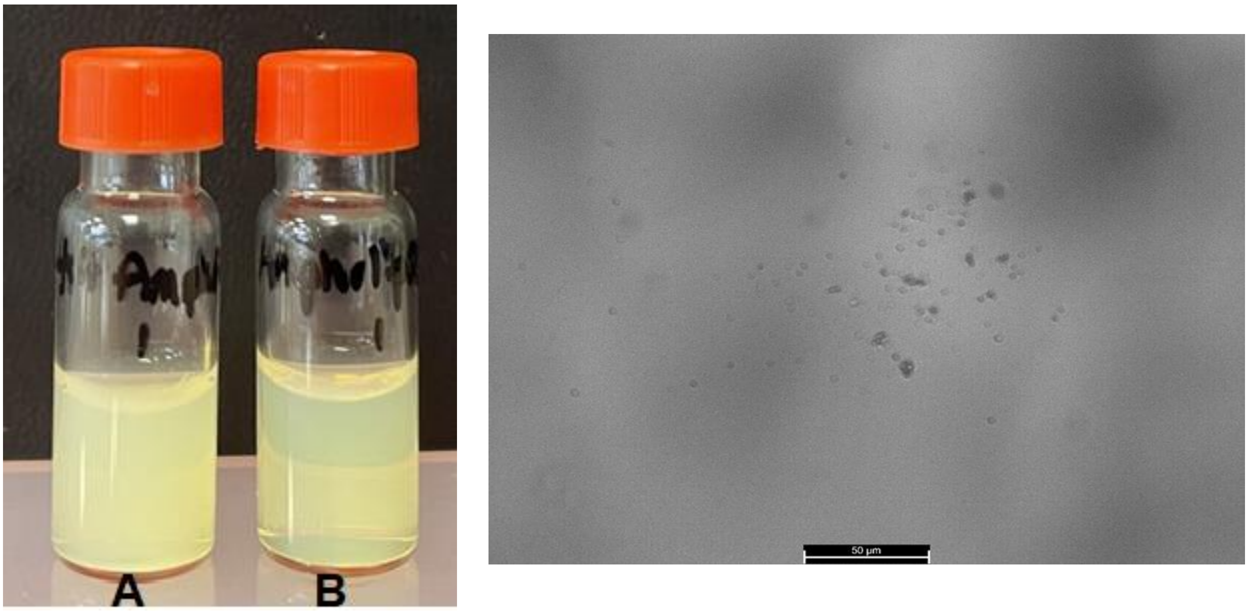

Figure S4 – Photograph (left) of the increase in opacity in the combination of caffeine citrate (20 mg/mL) and amphotericin liposomal 2mg/mL (A) compared to the control (B) (amphotericin + diluent); corresponding photomicrograph (right) (Leica MC190HD, objective x40) of test sample

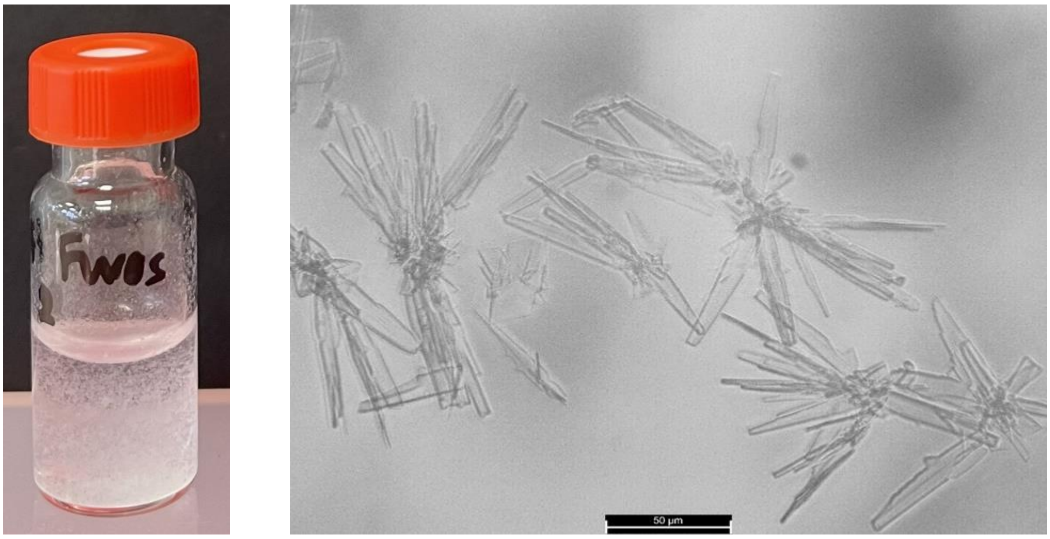

Figure S5 – Photograph (left) and corresponding photomicrograph (right) (Leica MC190HD, objective x40) of the white precipitate in the combination of caffeine citrate (20 mg/mL) and furosemide 1 mg/mL

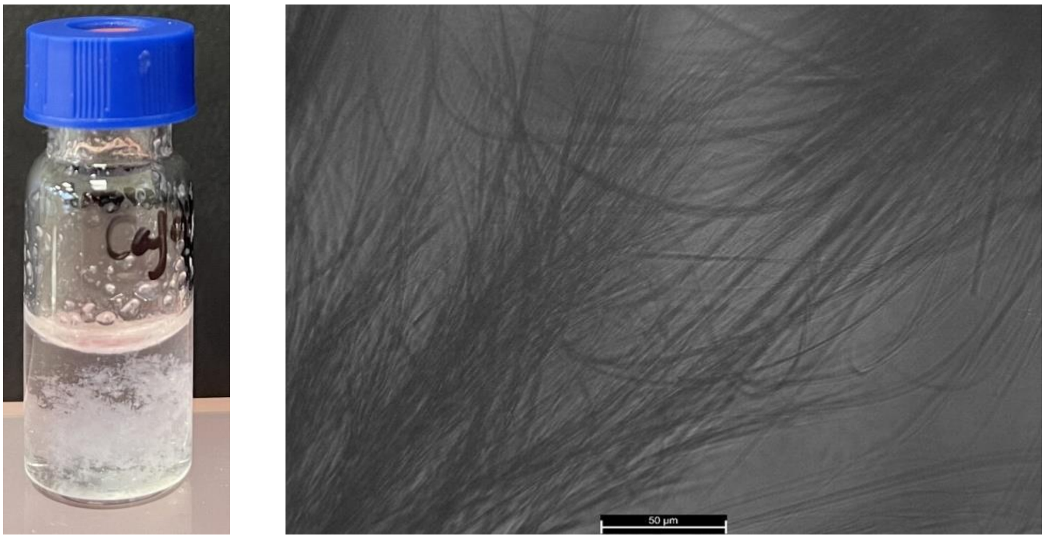

Figure S6 – Photograph (left) and corresponding photomicrograph (right) (Leica MC190HD, objective x40) of the white precipitate in the combination of caffeine citrate (20 mg/mL) and hydrocortisone 10 mg/mL

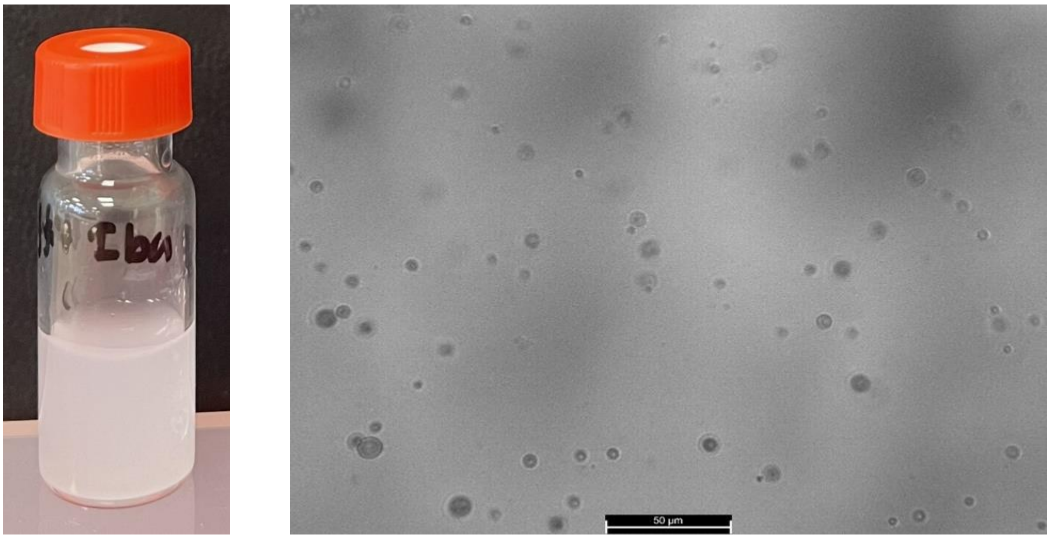

Figure S7 – Photograph (left) and corresponding photomicrograph (right) (Leica MC190HD, objective x40) of the milky turbidity in the combination of caffeine citrate (20 mg/mL) and ibuprofen 5 mg/mL

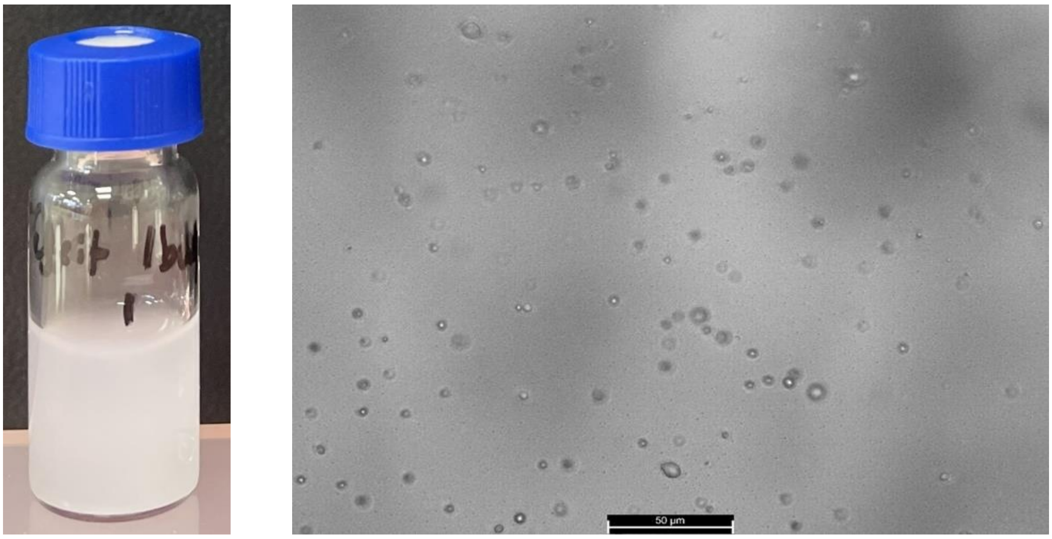

Figure S8 – Photograph (left) and corresponding photomicrograph (right) (Leica MC190HD, objective x40) of the milky turbidity in the combination of caffeine citrate (20 mg/mL) and ibuprofen lysine 4 mg/mL

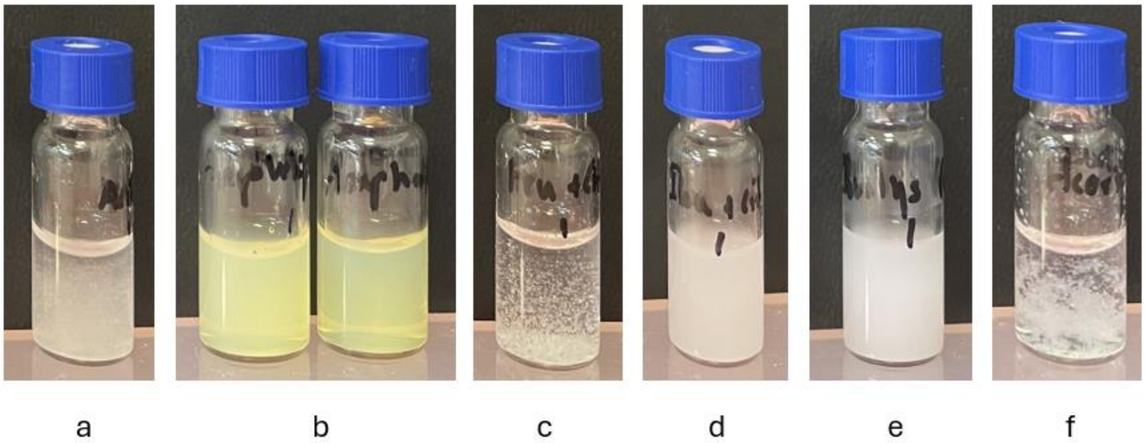

Figure S9 – Photographs of citrate buffer (pH 4.5) incompatibilities with drugs (a. aciclovir; b. amphotericin liposomal – test (left) and control (right); c. furosemide; d. ibuprofen; e. ibuprofen lysine; f. hydrocortisone)
